# Cardiometabolic Parameters and Transcription Factors in Rat Models of Prehypertension With or Without Hypertriglyceridemia: Focus on NRF2 and PPARα Gene Expression

**DOI:** 10.33549/physiolres.935717

**Published:** 2025-12-01

**Authors:** Aybuke BOZKURT, Michal KLUKNAVSKY, Peter BALIS, Andrea MICUROVA, Anjum ANJUM, Jana KOPINCOVA, Iveta BERNATOVA

**Affiliations:** 1 Centre of Experimental Medicine, Slovak Academy of Sciences, Institute of Normal and Pathological Physiology, Bratislava, Slovakia

**Keywords:** NRF2, PPARα, Vascular function, Liver, Heart

## Abstract

This study investigated selected cardiovascular, hepatic, and metabolic parameters, including *Nfe2l2*, *Hmox1* (an NRF2 target gene), and *Ppara* gene expression, in adult male normotensive Wistar-Kyoto (WKY), borderline hypertensive (BHR) and hereditary hypertriglyceridemic (HTG) rats. BHR and HTG rats exhibited increased blood pressure vs. WKY, but there were no differences in blood pressure of BHR and HTG rats. In contrast, HTG had elevated hematocrit, triacylglycerol levels, glycemia and atherogenic index of plasma, and decreased total cholesterol and HDL-cholesterol compared to BHR rats. In addition, nitric oxide synthase activity in the heart and liver was significantly reduced in HTG vs. BHR. Gene expressions of *Nfe2l2*, *Ppara*, and *Hmox1* were significantly elevated in the hearts of HTG rats compared to both WKY and BHR. In contrast, hepatic expression levels of *Nfe2l2* and *Hmox1* were significantly reduced in BHR and HTG compared to WKY, while *Ppara* expression in the liver was significantly reduced in HTG vs. both BHR and WKY. Vascular studies revealed that endothelium-dependent relaxation was reduced in HTG rats vs. BHR, suggesting a dominant effect of hypertriglyceridemia, while endothelium-independent relaxation was reduced in both HTG and BHR rats vs. WKY, suggesting a dominant effect of prehypertension in this vascular bed. Contraction responses were also more pronounced in HTG rats vs. BHR. Overall, this study showed that inherited hypertriglyceridemia (combined with prehypertension) alters vascular function and redox–metabolic balance in a tissue-dependent manner and represents a more significant cardiometabolic risk in later periods of life than prehypertension itself.

## Introduction

Although the number of adults with systolic blood pressure (BP) in the range of 120–139 mmHg and/or diastolic BP 80–89 mmHg (called also prehypertension, or borderline hypertension) is in range 15–23 % [1], the opinions on treatment of these patients to prevent the development of hypertension vary among the medical guidelines [2–4] and depend on evaluation of overall cardiometabolic risk of each individual. BP increase and the development of hypertension arises from both genetic predisposition and lifestyle factors, such as unhealthy diet or physical inactivity, and even genetically driven prehypertension can vary in its character and sensitivity to environmental stimuli. In some individuals, elevated BP develops without metabolic disorders, while in others, underlying metabolic alterations contribute to or accompany the rise in BP [4,5].

Borderline hypertensive rats (BHR), F1 offspring of spontaneously hypertensive rat (SHR) dams and Wistar-Kyoto (WKY) sires, display resting prehypertensive BP and serve to investigate early neurohumoral and vascular changes in preclinical studies [6]. Prehypertension also occurs in the hereditary hypertriglyceridemic (HTG) rats, which feature genetically determined hyper-triglyceridemia (without obesity) and vascular abnormalities [7,8].

In addition to inherited hypertriglyceridemia, it can also be diet-induced in rodents and humans. High intake of dietary fats or sugars (e.g., fructose) may lead to both hypertriglyceridemia and liver diseases. In diet-induced models, suppression of hepatic peroxisome proliferator-activated receptor alpha (PPARα), a master regulator of fatty acid β-oxidation and lipid metabolism, is commonly observed [9]. PPARα, encoded by *Ppara* gene, plays a protective role in metabolic syndrome and vascular dysfunction through improving lipid metabolism, reducing inflammation, and limiting reactive oxygen species (ROS) [10,11], while its downregulation is associated with hepatic steatosis, hypertriglyceridemia, and adverse cardiovascular outcomes [11]. In HTG rats, metabolic disturbances are also associated with oxidative and nitrosative stress [12].

In addition to PPARα, nutritional interventions and lifestyle modifications can modulate nuclear factor erythroid 2-related factor 2 (NRF2)-dependent antioxidant pathways, which play a crucial role in the cell’s ability to cope with oxidative stress and metabolic load [11,13]. NRF2, encoded by the *Nfe2l2* gene, regulates a broad spectrum of antioxidant response element-containing genes, involved in the modulation of redox balance, inflammation and lipid metabolism, which all may participate in the development of cardiometabolic diseases [14].

While several studies have examined vascular or metabolic markers in HTG rats, no previous work has systematically compared baseline, organ-specific mRNA expression of *Nfe2l2* and *Ppara* in HTG and BHR rats alongside the determination of nitric oxide (NO) production and vascular function in distinct arteries. Therefore, we aimed to compare these phenotypes in terms of: 1) Systemic parameters (biometrics and plasma markers including triacylglycerols (TAG), high-density lipoprotein-cholesterol (HDL-C), glucose, and atherogenic index (AI) of plasma), 2) Oxidative stress markers and NO synthase (NOS) activity in the heart, aorta, and liver, and 3) Vascular reactivity in the femoral and mesenteric arteries, assessing all endothelium-dependent and independent relaxations and contraction responses. 4) Most importantly, we investigated organ-specific gene expression of *Nfe2l2, Ppara*, and their common target gene *Hmox1* (encoding heme oxygenase-1, HO-1) in the heart and liver. We hypothesized that under a similar degree of prehypertension, hypertriglyceridemia imposes an additional burden on the endothelium, and the hypertriglyceridemia is associated with organ-specific alterations in *Ppara* and *Nfe2l2* gene expressions.

## Materials and Methods

### Animals and treatment

The experimental procedures used in this study were approved by the Ethics Committee of the Centre of Experimental Medicine, Slovak Academy of Sciences, Bratislava, Slovakia, and by the State Veterinary and Food Administration of the Slovak Republic (protocol code 4504-3/2024-220), in accordance with the European Union Directive 2010/63/EU. Breeding of all rats was conducted in the accredited animal facilities of the Slovak Academy of Sciences in Bratislava, Slovakia, in accordance with institutional guidelines in order to keep the same environmental background. Animals were housed under constant conditions of humidity (45–65 %), temperature (20–22 °C), and a 12-hour light/dark cycle. All rats had *ad libitum* access to laboratory rat chow (Altromin 1314P up to 8 weeks of age and Altromin 1324P from 8 weeks onward; Altromin International, Lage, Germany). Sixteen-week-old WKY (n = 10), BHR (n = 10), and HTG (n = 11) rats were anesthetized by inhalation with 3.5 % isoflurane vapour, and anaesthesia was maintained via a face mask. Before blood collection from the tail and thoracotomy, the depth of anaesthesia for surgical opening of the chest was verified by confirming the absence of the palpebral reflex in response to an interdigital pinch of the hind limb. Glycemia was measured in blood from the tail tip by a glucose meter (Accu-chek® Performa; Mannheim, Germany). Then, thoracotomy was performed to collect more blood from the right heart ventricle (RHV) as described below, followed by immediate decapitation.

The heart, aorta, liver, mesenteric, and femoral arteries were dissected. Tissue samples were rapidly weighed and processed for individual analyses. Samples were used fresh or snap-frozen in liquid nitrogen and stored at –80 °C until subsequent analyses.

### Blood pressure and biometric parameters determination

Systolic BP was measured by tail-cuff plethysmography using the CODA^®^ High Throughput System (CODA-HT4, Noninvasive Blood Pressure System, Kent Scientific Corporation, Torrington, CT, USA). All rats were handled and acclimated to the procedure in three independent sessions to minimize the effect of nonspecific stress. Rats were placed in restrainers and allowed to acclimate and preheat for about 10 minutes. The CODA^®^ system subsequently performed eight consecutive BP recordings, with the first three considered acclimatization trials and the following, at least three valid measurements, were used for the calculation of BP as the average value of valid measurements. BP was measured at the 12-week-old rats (basal) and then weekly for the next four weeks. After each BP measurement, the body weight (BW) of rats was recorded.

### Plasma biochemical analysis

Blood from the RHV was collected under permanent anesthesia into the 4 ml Li-heparinised (30 IU/mL) BD Vacutainer^®^ blood collection tubes, centrifuged (850 × g, 10 min, 4 °C; Centrifuge 5430 R, Eppendorf, Hamburg, Germany) and stored at −80 °C until further analysis. For biochemical assays, aliquoted plasma was thawed at room temperature, and 100 μl was loaded onto two reagent discs: General Chemistry IV Lyophilized Kit and Clinical Emergency Lyophilized Kit (Celercare^®^, MNCHIP Technologies, Tianjin, China). Plasma parameters were measured using a Celercare^®^ biochemical analyzer (MNCHIP Technologies, Tianjin, China). AI of plasma was calculated as log(TAG/HDL-C).

### Gene expression determination

The expression profiles of *Nfe2l2*, *Hmox1* and *Ppara* were analyzed in the heart and liver using real-time quantitative PCR. According to our preliminary study, 60S ribosomal protein L10a (*Rpl10a*) was used as a housekeeping gene in the left heart ventricle (LHV) and liver. The method and sequences of the forward and reverse primers of *Rpl10a*, *Nfe2l2*, and *Hmox1*, their amplicon sizes and melting temperatures were described in a previous study [15]. For determination of *Ppara* expression (NM_013196.1), forward primer: 5′-TGA ACA AAG ACG GGA TGC TGA T-3′ and reverse primer: 5′-TCA AAC TTG GGT TCC ATG ATG TC-3′ were used, which generate a 106 bp amplicon at melting temperature of 60 °C. Data were analyzed by the 2^−ΔΔCT^ method and expressed as fold change of the given gene.

### Measurement of conjugated dienes

Conjugated dienes (CD) were measured in 10 % tissue homogenates (w/v) of heart and liver as indicators of lipid peroxidation and oxidative damage as described previously by [16], with some modifications. Absorbance of the samples was measured at 233 nm. An extinction coefficient of 26,000 l/mol/cm was used to calculate the results, which were expressed in nanomoles of CD per gram of tissue (nmol/g).

### Nitric oxide synthase activity

Total NOS activity was determined in 20 % (w/v) homogenates of heart, aorta and liver by measuring [^3^H]-L-citrulline formation from [^3^H]-L-arginine (MP Biochemicals, Santa Ana, CA, USA), according to a protocol described previously [17]. Results are shown as picokatals per gram of protein (pkat/g protein). Protein content was determined using the Lowry method.

### Vascular reactivity

Vascular parameters were assessed in freshly isolated and connective tissue-free femoral and mesenteric arteries with intact endothelium. Arterial segments were inserted in myograph chambers (Dual Wire Myograph system 410A and Multi Myograph System 620M and 630MA, Danish Myo Technology A/S, Aarhus, Denmark), and vascular reactivity was continuously recorded using LabChart 8 software (ADInstruments NZ Limited, Dunedin, New Zealand). The chambers were filled with modified physiological saline solution (PSS) containing (in mmol/l): 119 NaCl, 4.7 KCl, 1.17 MgSO_4_·7H_2_O, 25 NaHCO_3_, 1.18 KH_2_PO_4_, 0.03 Na_2_EDTA, 2.5 CaCl_2_2H_2_O, and 5.5 glucose; maintained at 37 °C, pH 7.4, and oxygenated with 95 % O_2_/5 % CO_2_.

Both femoral and mesenteric arteries were evaluated for relaxant responses (Protocol 1) and contractile responses (Protocol 2). Following normalization and a 25-minute stabilization period, arterial viability was confirmed by exposure to a depolarizing solution (125 mmol/l K^+^, achieved by iso-osmotic substitution of NaCl with KCl in PSS) for 2 min. In Protocol 1, relaxation responses to acetylcholine (ACh, 10^−9^–10^−5^ mol/l) were assessed after preconstruction with serotonin (10^−6^ mol/l; Merck KGaA, Darmstadt, Germany) on the femoral artery (FA), and noradrenaline (NA, 10^−5^ mol/l) on the mesenteric artery (MA), as described previously [17–19]. Subsequently, after additional washout, vascular responses to cumulative concentrations of the exogenous NO donor sodium nitroprusside (SNP, 10^−9^–10^−5^ mol/l) were examined. In Protocol 2, cumulative concentration–response curves to NA (10^−9^–10^−4^ mol/l; Zentiva, Czech Republic) were recorded. All concentrations are expressed as final concentrations in the myograph chamber. Contractions were normalized to arterial segment length and expressed as (mN/mm). ACh- and SNP-induced relaxations are reported as relative (%) values of pre-contraction induced by an appropriate vasoconstricting substance.

### Statistical analysis

The data were expressed as means ± SEM. For the statistical evaluation of differences between groups, two-way analysis of variance (ANOVA) for repeated measures (BP, vascular functions) and a one-way ANOVA for other parameters with a Bonferroni post hoc test on ranks were used. Correlations between genes were analyzed by the Pearson correlation coefficient “r”. The differences between means were considered significant at p < 0.05. GraphPad Prism 8.0 (GraphPad Software; San Diego, USA), and Statistica 13.5 (StatSoft; Hamburg, Germany) were used for the statistical analyses.

## Results

### Cardiometabolic parameters

**Fig. 1:**
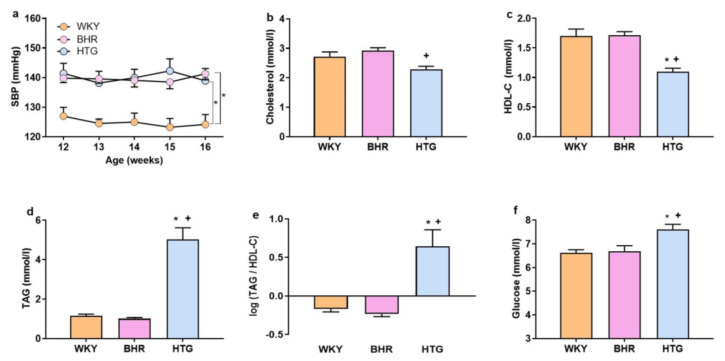
Cardiometabolic parameters – systolic BP (**a**), total plasma cholesterol (**b**), high-density lipoprotein-cholesterol (**c**), plasma triacylglycerols (**d**), atherogenic index of plasma (**e**), blood glucose (**f**). Abbreviations: BHR: borderline hypertensive rats, HDL-C: high-density lipoprotein-cholesterol, HTG: hypertriglyceridemic rats, SBP: systolic blood pressure, TAG: triacylglycerols, WKY: Wistar-Kyoto rats. The results are presented as the mean ± SEM of n = 10–11 rat/group, and ANOVA results are included in the text. *p < 0.05 vs. WKY, ^+^p < 0.05 vs. BHR.

Systolic BP was significantly higher in both BHR and HTG compared to WKY controls (F_(2,27)_ = 26,92, p < 0.00001, n = 30, main effect of strain) while no marked difference between BHR and HTG was recorded (Fig. 1a).

Metabolic profiling showed that total cholesterol was significantly reduced in HTG compared to BHR (F_(2,25)_ = 7.06, p < 0.004, n = 28; Fig. 1b), while HDL-C was significantly lower in HTG rats than in WKY and BHR (F_(2,25)_ = 23.32, p < 0.0001, n = 28; Fig. 1c). As expected, HTG rats had significantly elevated TAG levels (F_(2,25)_ = 117.38, p < 0.00001, n = 28), increased AI of plasma (F_(2,25)_ = 179.45, p < 0.00001, n = 28) and higher tail glucose concentrations (F_(2,24)_ = 8.33, p < 0.002, n = 27) compared to both WKY and BHR (Fig. 1d,e,f). These findings demonstrated that HTG rats exhibit dyslipidemia and hyperglycemia in addition to hypertension, distinguishing them from both BHR and WKY controls.

### Biometric parameters

BHR had significantly increased BW, higher relative kidney mass and hematocrit compared with WKY (Table 1). HTG rats had significantly higher BW than WKY, but lower relative mass of heart (both left and right ventricle mass), kidney and adrenal gland, together with elevated hematocrit. Relative heart, LHV and RHV masses and hematocrit of HTG were reduced when compared to BHR (Table 1).

### Plasma biochemic markers

Analysis of biochemical markers (Table 2) revealed significant changes in prehypertensive rats when compared to WKY. Both BHR and HTG rats showed an increase in total protein concentration and albumin, while HTG rats had an additional decrease in alkaline phosphatase (ALP) and creatinine (CREA) compared to both BHR and WKY rats, a decrease in alanine aminotransferase (ALT) compared to BHR and almost doubled α-hydroxybutyrate dehydrogenase when compared to WKY (Table 2). Additional changes in BHR included only an increase in CREA when compared to WKY (Table 2).

### Gene expression levels

**Fig. 2:**
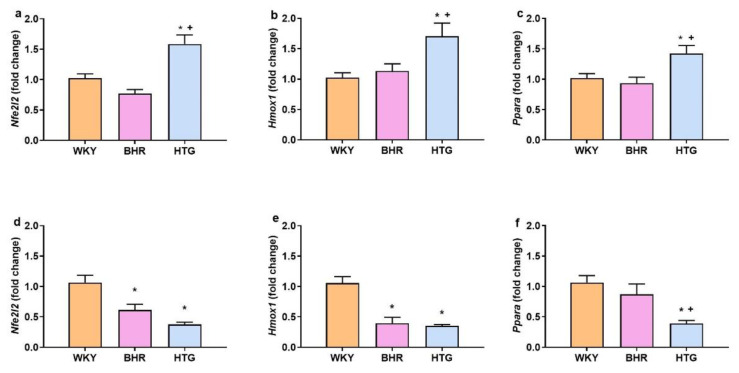
Gene expression levels of *Nfe2l2* (**a,d**), *Hmox1* (**b,e**), and *Ppara* (**c,f**) in heart (**a–c**) and liver (**d–f**). Abbreviations: BHR: borderline hypertensive rats; HTG: hypertriglyceridemic rats; WKY: Wistar-Kyoto rats. *p < 0.05 vs. WKY, ^+^p < 0.05 vs. BHR. The results are presented as the mean ± SEM of n = 10–11 rats/group. ANOVA results are included in the text.

In the heart, HTG rats had markedly upregulated expression levels of *Nfe2l2* (F_(2,24)_ = 15.04, p < 0.001, n = 27), *Hmox1* (F_(2,24)_ = 5.27, p < 0.02, n = 27), and *Ppara* (F_(2,24)_ = 6.14, p < 0.01, n = 27) compared to both WKY and BHR (Fig. 2a,b,c). In contrast, the liver expression of *Nfe2l2* (F_(2,26)_ = 15.71, p < 0.0001, n = 29) and *Hmox1* (F_(2,26)_ = 22.57, p < 0.0001, n = 29) was markedly reduced in both BHR and HTG relative to WKY (p < 0.05), with the lowest levels observed in HTG rats (p < 0.05; Fig. 2d,e). HTG rats showed also decreased *Ppara* (F_(2,26)_ = 9.21, p < 0.001, n = 29) expression in the liver compared to WKY (p < 0.05) and BHR (p < 0.05), while no significant difference was observed between WKY and BHR (Fig. 2f). In addition, there were positive correlations between *Nfe2l2* and *Ppara* expressions in both the heart (r = 0.84, p < 0.0001, n = 27) and liver (r = 0.47, p < 0.02, n = 29).

### Conjugated diene levels and nitric oxide production

**Fig. 3:**
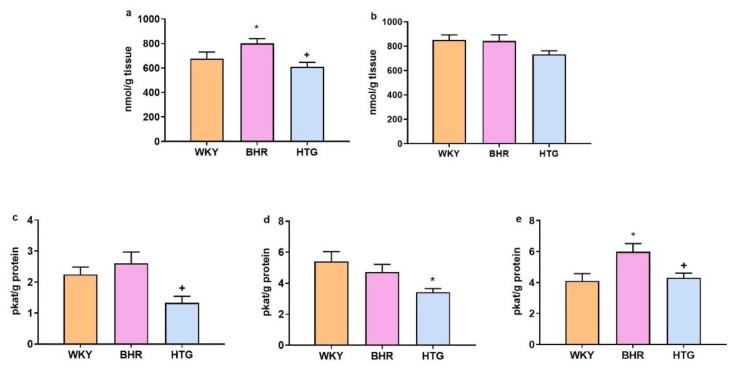
Conjugated dienes in heart (**a**) and liver (**b**), and nitric oxide synthase activity in the heart (**c**), aorta (**d**) and liver (**e**). Abbreviations: BHR: borderline hypertensive rats; HTG: hypertriglyceridemic rats; WKY: Wistar-Kyoto rats. The results are presented as the mean ± SEM of n = 10–11 rats/group. *p < 0.05 vs. WKY, ^+^p < 0.05 vs. BHR. ANOVA results are included in the text.

CD levels in the heart were significantly increased (F_(2,25)_ = 7.45, p < 0.01, n = 28) in BHR compared to WKY (p < 0.05), while HTG rats exhibited reduced levels relative to BHR (p < 0.05, Fig. 3a). In the liver, CD concentrations did not differ between the strains (F_(2,26)_ = 2.73, p = 0.08, n = 29; Fig. 3b).

NOS levels of HTG rats were significantly reduced in both the heart (F_(2,23)_ = 5.76, p < 0.01, n = 26) and the liver (F_(2,22)_ = 5.37, p < 0.02, n = 22) in comparison to BHR (p < 0.05; Fig. 3c, e), and in the aorta (F_(2,25)_ = 7.51, p < 0.04, n = 28) compared to WKY (p < 0.05; Fig. 3d). BHR showed increased NOS levels in the liver in comparison to WKY (p < 0.05; Fig. 3e)

### Vascular response of femoral and mesenteric arteries

Normalized diameter of the FA and MA did not differ significantly among the groups (Table 3). The replacement of PSS with high-potassium PSS (KPSS) elicited a pronounced contractile response due to membrane depolarization in both femoral and mesenteric arteries (Table 3). This response was significantly greater in BHR and HTG compared with WKY controls, as well as contractile responses of FA to serotonin, and MA to noradrenaline. Moreover, serotonin- and KPSS- induced pronounced response in FA of HTG when compared to BHR (Table 3).

**Fig. 4:**
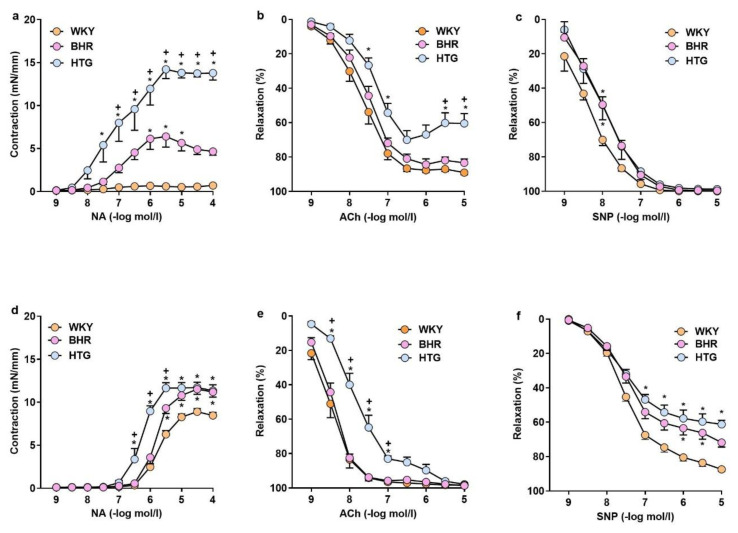
Vascular responses in the femoral and mesenteric arteries. Noradrenaline-induced contractions (**a,d**), acetylcholine-induced relaxation (**b,e**) and sodium nitroprusside-induced relaxation (**c,f**) in the femoral (**a–c**) and mesenteric arteries (**d–f**). Abbreviations: ACh: acetylcholine; BHR: borderline hypertensive rats; HTG: hypertriglyceridemic rats; NA: noradrenalin; SNP: sodium nitroprusside; WKY: Wistar-Kyoto rats. The results are presented as the mean ± SEM of n = 8–10 rats/group. ∗p < 0.05 vs. WKY, ^+^p < 0.05 vs. BHR

FA of normotensive WKY demonstrated the lowest NA-induced contraction (F_(20,240)_ = 19.00, p < 0.0001, n = 27; Fig. 4a) and maximal ACh-mediated endothelium-dependent relaxation (F_(16,192)_ = 4.14, p < 0.0001, n = 27, Fig. 4b). In HTG rats, NA-induced contraction was significantly greater compared to both WKY and BHR (Fig. 4a), while their endothelium-dependent relaxations to ACh were markedly impaired, which was seen also in comparison with BHR (Fig. 4b). SNP-induced endothelium-independent relaxations were reduced in the FA (F_(16,176)_ = 2.25, p < 0.0001, n = 25) and MA (F_(16,184)_ = 10.68, p < 0.0001, n = 26) in BHR and HTG compared to WKY (Fig. 4c, f).

## Discussion

Hypertension and its early stage, called prehypertension, is often accompanied by metabolic comorbidities, and – depending on genetics or environmental factors – dyslipidemia, (pre)diabetes, liver disorders, or endothelial dysfunction may occur. There is sustained interest in determining which factors are the cause of increased BP and which are the consequences [20]. However, in human studies, it is difficult to distinguish hemodynamic load (BP) from metabolic load (e,g., dyslipidemia), because these traits may track together [21]. Rodent models enable control of confounders (diet, age, BP) and determine the metabolic parameters such as oxidative stress, NO signaling, genetic alterations or vascular function at the level of specific organs and vascular beds [22].

To our best knowledge, this is the first study that directly compared BHR (prehypertensive without dyslipidemia) with the hereditary HTG rats (prehypertensive with dyslipidemia) at comparable systolic BP. By matching BP level, we could separate the “metabolic load” of hypertriglyceridemia from the “hemodynamic load” of prehypertension and examine various above-mentioned parameters. The main findings of this study are: HTG rats exhibit – compared to BHR – 1) more unfavourable systemic environment (alterations in lipid metabolism, elevated AI of plasma, higher glycemia) together with lower NO production, 2) more pronounced vasoconstrictor responses with impaired vasorelaxation, and 3) increased expression of cardiac *Nfe2l2*, *Hmox* and *Ppara* together with lower *Ppara* expression in the liver.

Both BHR and HTG rats exhibited higher systolic BP and greater BW than WKY, but as expected, only HTG showed elevated plasma TAG and higher glycemia. As HDL-C was reduced in HTG rats, this strain had also markedly elevated AI of plasma, confirming a higher risk of atherogenesis in the condition of hypertriglyceridemia [7,12]. Moreover, only HTG rats showed a reduction of the relative cardiac, renal and adrenal gland masses. HTG rats were previously reported to have an increase in relative heart weight [23], yet it is important to take notice of the control strain used for comparison. In this study, we used WKY, as males of this strain were used to produce BHR. However, unchanged total cholesterol, reduced HDL-C and lower relative heart weight were also reported in HTG male rats when compared to Wistar rats [12]. Previously, a detailed genetic linkage map of HTG﷓derived crosses have identified genetic determinants of organ weights (heart and kidney) that only partially track with BP [23], supporting the view that genetic hypertriglyceridemia alters organ structure (and supposedly also function) independently of BP. This view is further supported by an increase in relative kidney weight in HTG rats after an appropriate treatment of dyslipidemia [24] together with the fact that similar BP in BHR without dyslipidaemia led to elevated relative kidney mass compared to WKY in this study. Regarding LHV hypertrophy, its progress in BHR seems to be age-dependent; no differences in this parameter were found in young (7–9 weeks old) BHR [6,25], while an elevated LHV-to-BW ratio was found in 12-week-old BHR [26]. However, ageing might be associated with a decrease in this parameter, as it was found previously in WKY and male SHR [27].

Investigation of haematocrit and biochemical markers showed higher haematocrit, total protein and albumin in both BHR and HTG rats vs. WKY, while globulins were selectively increased in HTG rats. This finding is compatible with low-grade inflammation that has been documented in HTG rats [28], which can reflect changes in blood viscosity that had been associated with BP increase [29]. By contrast, hepatobiliary enzymes were not elevated in HTG rats, which is different from diet﷓induced hypertriglyceridemia or non-alcoholic fatty liver disease (NAFLD) models, where ALT, aspartate aminotransferase, and ALP typically rise [30], indicating that genetic HTG may not be associated with hepatocyte damage.

However, we found considerable alterations in the gene expressions of prehypertensive rats, which were tissue-dependent. In the liver, both BHR and HTG rats showed reduced *Nfe2l2* and *Hmox1* mRNA compared to WKY. Changes in *Hmox1*, which are in agreement with changes in *Nfe2l2* expression, suggest that NRF2 signaling is decreased in the liver of prehypertensive rats, as *Hmox1* gene is a canonical NRF2 target [31]. In addition, HTG rats demonstrated a selective down﷓regulation of *Ppara* vs. both WKY and BHR, and there were significant positive correlations between *Nfe2l2* and *Ppara* in both tissues investigated. As PPARα is functionally linked to NRF2 signaling and, among others, it is associated with balancing circulating free fatty acids and triacylglycerol levels [10,32], decreased hepatic expression of *Ppara* seems to be an important mechanism involved in the development of hypertriglyceridemia in this strain. Reduced *Ppara* in the liver of HTG rats was found previously compared to Wistar rats; however, *Nfe2l2* mRNA was elevated in contrast to our study [33]. In addition, *Ppara* agonists, which lowered TAG and elevated *Ppara* expression, were shown to reduce *Nfe2l2* mRNA in HTG rats [34]. Thus, the relation of *Ppara* and *Nfe2l2* expression in basal and PPARα (or NRF2) stimulated conditions needs further investigation.

Interestingly, findings in the heart were different from those in the liver. HTG rats in our study – despite BP matching to BHR – showed lower myocardial lipid peroxidation and smaller relative cardiac mass, together with higher cardiac expression of *Nfe2l2*, *Hmox1* and *Ppara*. We interpret elevated gene expressions as a compensatory redox–metabolic response that reduces lipotoxic injury during the systemic TAG overload and cardiac NO deficiency. Activation of PPARα was also associated with attenuation of cardiac remodelling during pressure overload, suggesting the protective role of PPARα on the heart [35]. Taken together, these data suggest that cardiac NRF2/PPARα activation can result in reduced lipid peroxidation (vs. BHR), and, presumably to activation of cardioprotective mechanisms.

Regarding NO production, our finding of lower hepatic NOS activity in HTG rats aligns with the redox milieu: at a weakened NRF2/HO-1 pathway, excessive ROS can quench NO, resulting in peroxynitrite formation, uncoupled endothelial NOS (eNOS), and diminish vascular NO signaling. Studies that investigated fructose-induced hypertriglyceridemia reported nitroxidative stress with increased inducible NOS and protein nitration in models [36], while reviews highlight a protective role of hepatocellular eNOS in NAFLD – together supporting a redox﷓driven NO deficit when antioxidant defenses and PPARα﷓dependent lipid oxidation are compromised [37]. Reduced NO production in the heart and aorta was also found compared to Wistar rats [38,39], although these changes were less pronounced compared to WKY rats in our study.

Regarding vascular function, in our study, contraction responses were enhanced in FA and MA, and ACh﷓induced relaxation was depressed in HTG rats more than in BHR in both arteries investigated. SNP-induced endothelium﷓independent relaxation was preserved in the FA but reduced in the MA (in both strains), indicating regional differences in the downstream sensitivity of arteries to NO. Endothelial dysfunction in HTG rats can result from ultrastructural abnormalities of the intima as found in the aorta [40] or increased sympathetic tone [41]. In addition, hypertriglyceridemic serum alone attenuated ACh responses and increased contractility in isolated arteries, confirming the negative effect of circulating metabolic factors on endothelial function [42]. Furthermore, selective hypertriglyceridemia reduced hindlimb flow responses to ACh *in vivo* [43], which is in line with findings of endothelial dysfunction in the femoral artery in this study. Thus, our study showed that genetic hypertriglyceridemia was associated with lowered NOS activity in large vessels (as we observed) and blunted endothelium-dependent relaxation. On the other hand, preserved SNP-induced responses in the FA suggest intact soluble guanylate cyclase/cyclic guanosine monophosphate signaling in conduit arteries, whereas the depression of SNP-induced responses in the MA indicates impairment in the resistance vascular bed. Reduced endothelium-independent relaxation in the MA seems to be associated with prehypertension, as it was comparable in BHR and HTG rats. The findings of worsened endothelium-dependent relaxation and elevated contraction responses in HTG rats compared with BHR confirm high vascular risk in hypertriglyceridemic individuals, which is also in agreement with remarkably elevated AI of plasma in HTG. In addition, the upregulation of *Nfe2l2* and *Ppara* observed in the hearts of HTG rats contrasts with their hepatic downregulation, indicating organ-specific adaptations to metabolic stress, confirming our hypothesis.

In conclusion, HTG rats exhibited a clear metabolic burden, characterized, in addition to hypertriglyceridemia, by higher glycemia, an elevated AI of plasma, and altered NO production in the liver and cardiovascular system. Vascular studies revealed that endothelium-dependent relaxation was reduced in HTG rats vs. BHR, suggesting a dominant effect of hypertriglyceridemia, while endothelium-independent relaxation was reduced in both HTG and BHR rats, suggesting a dominant effect of prehypertension in this vascular bed. Contraction responses were also more pronounced in HTG rats vs. BHR. In the liver, downregulation of PPARα and NRF2 signaling suggests compromised lipid metabolism and antioxidant defense system, whereas their upregulation in the heart may reflect a compensatory adaptation to oxidative and metabolic stress. Overall, this study showed that inherited hypertriglyceridemia (combined with prehypertension) alters vascular function and redox–metabolic balance in a tissue-dependent manner and represents a more significant cardiometabolic risk in later periods of life than prehypertension itself.

## Figures and Tables

**Table 1:** Biometric parameters

Parameter	WKY	BHR	HTG	*ANOVA*
*Body weight (g)*	326.5 ± 4.60	362.1 ± 4.20*	374.4 ± 7.06*	F_(2,26)_ = 20.91 p < 0.001
*Heart/BW (mg/g)*	3.28 ± 0.05	3.34 ± 0.04	2.38 ± 0.04*+	F_(2,26)_ = 138.5 p < 0.0001
*LHV/BW (mg/g)*	1.76 ± 0.05	1.63 ± 0.03	1.21 ± 0.04*+	F_(2,26)_ = 48.0 p < 0.0001
*RHV/BW (mg/g)*	0.63 ± 0.03	0.57 ± 0.01	0.42 ± 0.01*+	F_(2,26)_ = 28.3 p < 0.0001
*Liver/BW (mg/g)*	37.67 ± 0.75	35.384 ± 0.48	37.35 ± 1.20	F_(2,26)_ = 1.165 p = 0.32
*Kidney/BW (mg/g)*	6.71 ± 0.11	7.13 ± 0.07*	5.09 ± 0.05*+	F_(2,26)_ = 162.40 p < 0.0001
*Adrenal gl./BW (μg/g)*	145.0 ± 3.50	140.18 ± 3.93	128.34 ± 4.26*	F_(2,26)_ = 4.93 p < 0.02
*Hematocrit (volume %)*	42.44 ± 0.34	43.78 ± 0.22*	45.43 ± 0.20*+	F_(2,22)_ = 24.90 p < 0.0001

Abbreviations: Adrenal gl.: adrenal gland, BHR: borderline hypertensive rats, BW: body weight, HTG: hypertriglyceridemic rats, LHV: left heart ventricle, RHV: right heart ventricle, WKY: Wistar-Kyoto rats. The results are presented as the mean ± SEM of n = 10–11 rat/group.

* p < 0.05 vs. WKY;

+ < 0.05 vs. BHR.

**Table 2:** Biochemic markers

Parameter	WKY	BHR	HTG	*ANOVA*
*Total protein (g/l)*	58.70 ± 2.93	66.76 ± 0.49*	69.43 ± 0.96*	F_(2,25)_ = 10.08 p < .001
*Albumin (g/l)*	34.44 ± 1.88	40.50 ± 0.26*	40.19 ± 0.43*	F_(2,25)_ = 9.59 p < 0.001
*Globulin (g/l)*	23.70 ± 1.07	26.55 ± 0.33	29.24 ± 0.63*+	F_(2,25)_ = 14.43 p < 0.001
*ALT (U/l)*	96.55 ± 7.21	111.00 ± 6.63	83.20 ± 2.65+	F_(2,25)_ = 6.06 p < 0.01
*AST (U/l)*	125.56 ± 13.04	158.33 ± 11.44	139.40 ± 11.33	F_(2,25)_ = 1.83 p = 0.18
*ALP (U/l)*	289.89 ± 17.75	286.67 ± 382	177.00 ± 8.81*+	F_(2,25)_ = 32.39 p < 0.0001
*CREA (μmol/l)*	71.88 ± 3.45	84.88 ± 3.44*	59.90 ± 2.75*+	F_(2,25)_ = 15.40 p < 0.0001
*UREA (mmol/l)*	7.48 ± 0.59	6.89 ± 0.11	7.56 ± 0.27	F_(2,25)_ = 0.69 p = 0.51
*LDH (U/l)*	217.00 ± 26.11	278.25 ± 51.50	278.30 ± 31.49	F_(2,23)_ = 0.87 p = 0.434
*αHBDH (U/l)*	64.25 ± 7.43	92.12 ± 14.59	115.20 ± 14.73*	F_(2,23)_ = 3.84 p < 0.036
*K* *+* * (mmol/l)*	5.73 ± 0.32	5.82 ± 0.32	5.01 ± 0.17	F_(2,23)_ = 2.98 p = 0.071
*Cl* *^−^* * (mmol/l)*	99.66 ± 4.2	106.0 ± 0.82	106.81 ± 0.91	F_(2,23)_ = 2.10 p = 0.146
*Na* *+* * (mmol/l)*	139.62 ± 7.57	148.75 ± 1.43	147.00 ± 0.78	F_(2,23)_ = 1.30 p = 0.29
*CO* *_2_* * (mmol/l)*	27.89 ± 1.43	28.42 ± 0.54	27.27 ± 0.61	F_(2,23)_ = 0.42 p = 0.662

Abbreviations: ALP: alkaline phosphatase, ALT: alanine aminotransferase, AST: aspartate aminotransferase, BHR: borderline hypertensive rats, CREA: creatinine, αHBDH: α-hydroxybutyrate dehydrogenase, HTG: hypertriglyceridemic rats, LDH: lactate dehydrogenase, U: enzyme activity unit, WKY: Wistar-Kyoto rats. The results are presented as the mean ± SEM of n = 10–11 rat/group.

* p < 0.05 vs. WKY;

+ p< 0.05 vs. BHR.

**Table 3:** Vascular parameters of the femoral and mesenteric arteries.

Femoral artery (FA)	WKY n = 10	BHR n = 9	HTG n = 8	*ANOVA*
*Normalized diameter (μm)*	824.97 ± 4.46	818.14 ± 7.42	806.13 ± 7.69	F_(2,25)_ = 2.11 p = 0.14
*KPSS* *_max_* * (mN/mm)*	9.66 ± 0.32	11.26 ± 0.38*	13.11 ± 0.48*^+^	F_(2,25)_ = 19.22 p < 0.0001
*Ser* *_max_* * (mN/mm)*	12.38 ± 0.28	15.14 ± 0.30*	16.74 ± 0.45*^+^	F_(2,25)_ = 41.49 p < 0.0001

**Mesenteric artery (MA)**	**WKYn = 10**	**BHRn = 9**	**HTGn = 8**	***ANOVA***

*Normalized diameter (μm)*	398.26 ± 8.86	405.65 ± 7.22	372.26 ± 12.97	F_(2,24)_ = 3.06 p = 0.07
*KPSS* *_max_* * (mN/mm)*	8.28 ± 0.33	10.90 ± 0.36*	10.51 ± 0.58*	F_(2,24)_ = 12.15 p < 0.002
*NA* *_max_* * (mN/mm)*	8.77 ± 0.41	12.75 ± 0.39*	12.9 ± 0.71*	F_(2,24)_ = 22.64 p < 0.0001

Abbreviations: BHR: borderline hypertensive rats; HTG: hypertriglyceridemic rats, KPSS_max_: maximum vascular contraction induced by high-potassium physiological salt solution, Ser_max_: maximum vascular contration induced by serotonin; NA_max_: maximum vascular contraction induced by noradrenaline; WKY: Wistar-Kyoto rats. The results are presented as the mean ± SEM of n = 8–10 rats/group.

* p < 0.05 vs. WKY.
